# α-Synuclein-mediated neurodegeneration in Dementia with Lewy bodies: the pathobiology of a paradox

**DOI:** 10.1186/s13578-021-00709-y

**Published:** 2021-11-19

**Authors:** Christopher Simon, Tomoko Soga, Hirotaka James Okano, Ishwar Parhar

**Affiliations:** 1 Brain Research Institute, Jeffrey Cheah School of Medicine and Health Sciences, Monash University Malaysia, Bandar Sunway, Selangor, Malaysia; 2 Division of Regenerative Medicine, Research Center for Medical Sciences, The Jikei University School of Medicine, Tokyo, Japan

**Keywords:** Alzheimer’s disease, Parkinson’s disease, Braak hypothesis, Oligomers, Fibrils

## Abstract

Dementia with Lewy bodies (DLB) is epitomized by the pathognomonic manifestation of α-synuclein-laden Lewy bodies within selectively vulnerable neurons in the brain. By virtue of prion-like inheritance, the α-synuclein protein inexorably undergoes extensive conformational metamorphoses and culminate in the form of fibrillar polymorphs, instigating calamitous damage to the brain’s neuropsychological networks. This epiphenomenon is nebulous, however, by lingering uncertainty over the quasi “pathogenic” behavior of α-synuclein conformers in DLB pathobiology. Despite numerous attempts, a monolithic “α-synuclein” paradigm that is able to untangle the enigma enshrouding the clinicopathological spectrum of DLB has failed to emanate. In this article, we review conceptual frameworks of α-synuclein dependent cell-autonomous and non-autonomous mechanisms that are likely to facilitate the transneuronal spread of degeneration through the neuraxis. In particular, we describe how the progressive demise of susceptible neurons may evolve from cellular derangements perpetrated by α-synuclein misfolding and aggregation. Where pertinent, we show how these bona fide mechanisms may mutually accentuate α-synuclein-mediated neurodegeneration in the DLB brain.

## Background

The emergence of “Dementia with Lewy bodies” (DLB), as a notion, was entirely based on geographically-defined schools of thought and revolutionary pathological analyses at a time when neurogenetics was beginning to contribute to our understanding of this understudied yet devastating condition [1, 2]. Once reputed to be a clinical syndrome depicted in isolated case reports, DLB has been purported to be the second most prevalent neuropathologic substrate of dementia [3–5] characterized by visual hallucinations, parkinsonism, and a fluctuating clinical course in older individuals [6, 7]. Contingent upon the nature of the population probed and the diagnostic criteria adapted, estimates from community-based clinical cohorts have been as high as one-quarter of all demented cases over the age of 65 [5, 8], parallel to approximations from post-mortem successions which have spanned between 15 and 25% [3, 9]. Reminiscing over 50 years of DLB research, the first inroads of this remarkable evolution commenced with James Parkinson’s intricate narrative of a Lewy body-like disorder vaguely identified as Shaking Palsy [10], which is what we now call Parkinson’s disease (PD) [11]. Despite the comprehensive portrayal of psychomotor manifestations, the unprecedented monograph made no allusion to the pathological and cognitive aspects of the disease, ultimately concluding that the intellect was ‘unharmed’ [10]. It was not until 1912 that Friedrich Heinrich Lewy began to explicate Parkinson’s call for pathological clarification [12] when he showcased the first depiction of eosinophilic intracytoplasmic and intraneuritic inclusions in the substantia innominata and dorsal vagal nuclei of autopsied PD brains [1, 13]. In less than a decade later, the term “Lewy body” (corps de Lewy) would go on to be enunciated for the first time by Konstantin Nikolaevich Tretiakoff [14], who documented the occurrence of analogous proteinaceous inclusions in the post-mortem substantia nigra of parkinsonian subjects [1, 14]. After a hiatus lasting more than 50 years, Kenji Kosaka went on to propose a new disease entity which he collectively termed “diffuse Lewy body disease” following his discovery of the first autopsy case with ‘Lewy-like-bodies’ pathology [15, 16]. The clinicopathological manifestations of individuals with diffuse cortical Lewy body disease were elucidated by Gibb and colleagues 3 years later [17, 18], and in 1992, the first operational consensus of senile dementia of Lewy body type (SDLT) was defined [19]. In spite of these early circumstantial nomenclatures and metaphors, the currently accepted conceptual framework formulated for early and prodromal diagnosis of DLB was first published in 1996 by the internationally renowned Consortium on DLB [6, 20].

Hitherto, delineating the nosological horizon of DLB diagnoses with respect to both Alzheimer’s disease (AD) and Parkinson’s disease dementia (PDD) has been an obstinate dilemma that reflects the fundamental pathobiology of the disease. Although DLB was first recognized as a rare pathological malady [21], it was gradually comprehended that α-synuclein-immunoreactive Lewy bodies [22] were recurrent ramifications in dementia cases with plaque pathology [23–26] and in PDD, specifically in cases where the latter had advanced to encompass cognitive deficits [27, 28]. Be that as it may, whether these juxta-nuclear inclusions represent an adaptive manifestation of a cytoprotective response or a defective self-preservation mechanism at the cellular level has yet to be singled out. Over the years, semi-quantitative assessments of autopsy-proven cases evaluating the neuroanatomical vulnerability between Lewy body biogenesis and DLB clinical phenotypes have reported contradictory repercussions. In a fraction of DLB cases, the approximated amount of cortical Lewy bodies in relation to total neuronal count correlates faintly with the severity and duration of cognitive and neuropsychiatric symptoms [29–32]. On the other hand, several lines of evidence have disparately verified a pronounced correlation between neocortical Lewy body densities and the clinical indices of dementia severity accompanying long-standing DLB [33, 34]. This dichotomy of regional pathology was also ascertained in neurochemical profiles of DLB patients [35, 36], where paradoxical alterations in neurochemical levels did not always correlate with cognitive domains [37]. Although these traits persist at the core of DLB, our conceptualization of the disease is evolving, with mounting evidence that α-synuclein-mediated synaptic aberrances discerned at predilection sites of neuronal depletion and Lewy body morphogenesis [38, 39] precede the clinical onset of DLB [40–44]. In post-mortem DLB brain samples, the subcellular localization of hyper-phosphorylated [45–47], detergent-insoluble [48, 49], and proteinase K-resistant [40, 50] α-synuclein aggregates in presynaptic-enriched fractions was affiliated with profound synaptic degeneration [51, 52]. These provocative findings are reminiscent of the observation that Lewy bodies are formed by microtubule-dependent cytoprotective aggresomes that sequester and compartmentalize synaptosomal α-synuclein aggregates from the neuronal periphery [53, 54]. Mechanistically, the former phenomenon is somewhat anecdotal and has been disputed, yet it would be thoroughly congruous with the supposition that DLB is a primary synaptopathy.

Emerging parallels posit that the intermolecular dynamics underlying the transformation of α-synuclein from its benign soluble state into fibrillized pathogenic inclusions entails a complex series of modifications that extend beyond the process of inclusion formation. According to the most simplistic viewpoint, physicochemical factors that enhance α-synuclein levels, catalyze its misfolding, or incite its translocation from its physiological presynaptic organization could possibly provoke de novo LB pathology [23, 55–62]. Supporting a role in causation, DLB-associated hereditary mutations in α-synuclein were also unambiguously consistent with the high densities of Lewy bodies in the subcortical nuclei, parahippocampus, amygdala, and cortex. The fact that this notation holds true in familial DLB kindreds with α-synuclein locus multiplications shows that α-synuclein expression is integral in determining whether the gradual degeneration of susceptible neurons prevails [63–66]. Yet, despite being implicated across a continuum of clinical phenomenologies [67], the pathogenic behavior of α-synuclein species in the DLB brain remains inadequately axiomatic. Recent reports connote that the insoluble filamentous α-synuclein in LBs may not be the prime neurotoxic culprit underlying neuronal dyshomeostasis and clinical variability. In fact, complementary cellular experiments have broadly featured the noxious insults of soluble α-synuclein oligomers as an adjuvant to the spreading of Lewy pathology [68, 69]. Thus, in this review, we explicatively explore experimental contexts highlighting the seminal contributions of α-synuclein conformers to the pathogenesis of DLB as an underappreciated pathological paradox of the DLB pathocascade. In particular, we assimilate and delineate the molecular prototypes that have led to new insights into understanding the mechanistic role that α-synuclein abnormalities dictate during their subsequent propagation in the DLB brain. Such a deliberation is, of necessity, selective rather than exhaustive, and its perspective may exhilarate the development of a tantalizing α-synuclein-centric therapeutic armamentarium capable of addressing the diverse biological defects underlying DLB pathophysiology.

## Dementia with Lewy bodies: a pathonosological dilemma

Notwithstanding the designation it has attained in the classification of nosologists [6], DLB is an enigmatic clinical entity of a nature highly insidious since its clinicopathological overlaps with AD and PDD are clearly arbitrary and arduous to define [70–72]. In recent years, there have been considerable attempts to demarcate the neuropsychological features that distinctively characterize the disorder and distinguish it from AD and PDD [73–76]. These methodical proceedings have been corroborated by retrospective neuropathological analyses that have identified anomalous patterns of brain aberrations in DLB [22, 77–83]. Given the well-defined criteria, patients with DLB have been shown to display subcortical pathological similarities to that of PDD, but with fewer LBs and a milder degree of neuronal cell depletion in the substantia nigra and other brainstem nuclei (e.g., locus coeruleus and dorsal vagal nucleus) [71, 81, 84, 85]. Accordingly, diffusely scattered cortical Lewy bodies were also primarily concentrated in the neocortex, transentorhinal cortex, entorhinal cortex, hippocampus, amygdala, insula, and cingulate [86–88]. Conversely, the pursuit for neuropsychological features that may potentially distinguish DLB from AD was obfuscated by the fact that a substantial majority of DLB patients have concomitant AD pathology (i.e., neurofibrillary tangles, neuritic plaques) that is adequate to meet the neuropathological diagnostic criteria for AD [20, 89, 90]. This pattern of AD pathology was ascertained indistinguishably throughout the DLB brain and was, for the most part, identical to ‘pure’ AD as it plagued cortical areas that were afflicted by Lewy pathology [91]. Adding to the intricacy of diagnostic considerations, both patients with DLB and pure AD typically displayed widespread deficiencies in cortical choline acetyltransferase (ChAT) levels, although the degree of reduction was considerably greater in DLB [92–94]. Unlike their AD counterparts, DLB patients are further characterized by the dopaminergic deafferentation of the striatum due to selective degeneration of pigmented substantia nigra neurons [95, 96]. Given the resemblances in the nature and dissemination of neuropathological alterations in DLB and AD, it is not surprising that both entities are at first classified by the insidious onset of cognitive deterioration with no other conspicuous neurological abnormalities [6, 70, 97–99]. Early-onset neuropsychiatric features such as memory deficits, delusions, and delusional misidentifications frequently predate cognitive impairments, and with time, patients inexorably progress to severe dementia. Indeed, the clinical manifestations are so comparable that DLB patients are habitually misdiagnosed as having possible or probable AD [89, 100]. There are, however, certain clinical features that manifest with a greater incidence in patients with DLB as opposed to those with pure AD [101]. These unequivocal attributes consist of spontaneous motor characteristics of parkinsonism, history of rapid eye movement (REM) sleep behavior disorder (RBD), recurring vivid visual hallucinations, and fluctuating cognition with marked disparities in attention or awareness [6, 19, 20, 89, 100, 102]. Despite this, the prevailing commonalities in dementia syndromes engendered by DLB and AD remain undisputed, which raises the question of whether Lewy body pathology contributes eminently to the syndrome beyond the realms of AD pathology [34, 103]. The relevance of Lewy-related pathology to the pathogenic mechanisms accountable for eliciting the wide phenotypic spectrum of DLB is still controversial. Numerous clinicopathological correlates have failed to associate LB density with the severity of parkinsonism, recurrent falls, cognitive fluctuations, visual hallucinations, delusions, age of onset, and disease duration [31, 88, 104–106].

Perceived by some as the “Holy Grail of DLB,” Lewy bodies (LBs) are intracytoplasmic eosinophilic inclusions predominantly expressed in selectively vulnerable neuronal perikarya of the DLB brain [5, 107, 108]. While the majority of LBs are single and spherical in shape and morphology, a substantial fraction of neurons is almost invariably accompanied by multiple or pleomorphic LBs [17, 91, 109]. From an ultrastructural perspective, classic brainstem LBs are marked by the occurrence of filamentous and amorphous granular material, with an argyrophilic core that lacks discernible detail and a peripheral halo that has radially arranged 10 nm filaments [110–115]. In specific vicinities of the brain, for instance, the dorsal vagal motor nucleus and the basal nucleus of Meynert, distribution of analogous inclusions are detected within neuronal processes and are often denominated as ‘intraneuritic’ LBs [15, 21, 116]. Intraneuritic LBs are presumably identified via immunohistochemical characterization [15] and should be discerned from Lewy neurites (LNs) [117], which are sometimes capricious and not distinguishable through conventional histopathology [118, 119]. The thread-like LNs were first detected in the hippocampus [118] but are also abundant in various brain structures, including the cingulate gyrus, entorhinal cortex, amygdala, and basal ganglia [120, 121]. Remarkably, there is a rapid expansion in the relationship of neuritic pathology to cognitive severity in DLB, especially since the constellation of neurons affected with LBs can be relatively small in a minority of cases [34, 122, 123]. In addition to the well-circumscribed hyaline inclusions, some neuronal populations develop lesions that bear a striking resemblance to the antigenic determinants of LBs [124] but are poorly circumscribed and easily disregarded in routine histological preparations [91]. These pale-staining eosinophilic lesions are typified as pre-Lewy bodies or pale bodies and are substantially more common in pigmented nigral neurons [91, 125]. Contrariwise, similar inclusions within cortical neurons are referred to as cortical LBs [21, 126], which tend to be confined to pyramidal and small non-pyramidal neurons in lower cortical layers (layers V and VI of the neocortex) [17, 127]. At the immunoelectron microscopic level, cortical LBs and LNs appear to be made up of haphazardly arranged 10 nm filaments in the halo but lack a distinctive dense core with a matted network of filaments [118]. Occasionally, however, a spectrum of cortical LBs consume a hyaline appearance similar to brainstem LBs [17, 119, 128, 129].

Revolutionary paradigmatic advances in biochemical and immunohistochemical techniques further illustrated that the primary structural constituent of LB-laden neurons is α-synuclein [23, 25, 130–133]. The α-synuclein deposited within LBs and LNs are conventionally detergent-insoluble [23] and are subjected to a plethora of post-translational modifications, with N- and C-terminal truncations, SUMOylation, nitration, ubiquitination, and Serine 129 phosphorylation being the predominant modifications [47, 55–57, 59, 134]. In fact, the immunostaining of human post-mortem DLB brains with serine-129 phosphorylated α-synuclein antibodies displayed a staggeringly profound accumulation of α-synuclein in LBs and LNs, in contrast to the visualization by phosphorylation-independent antibodies. Although there was some initial skepticism, deposits of Lewy threads and Lewy dots were comparably immune-positive for this post-translationally modified form of α-synuclein [135, 136]. As such, it is enticing to postulate that cell types in specific brain regions may well accumulate discretely modified forms of α-synuclein, which could potentially have insinuations in deciphering the clinico-pathological subtypes of DLB. Coincidently, a detailed molecular dissection of LB biogenesis revealed that ubiquitin, a small heat-shock protein involved in energy-dependent protein degradation, and the ubiquitin-binding protein p62, manifest in most classical and cortical LBs [137, 138]. Thus far, a repertoire of proteins implicated across various cellular programs has been detected within the confines of LBs via immunohistochemical quantification. Initially, an antibody-based profiling approach unveiled proteins that were engaged in lysosomal and proteasomal degradation, cell cycle regulation, and mitochondrial function. Alternatively, an in-depth proteomic investigation of LBs purified from DLB cortices exposed proteins that had relevance for oxidative stress, signal transduction and apoptosis, synaptic transmission and vesicular transport, folding and intracellular trafficking, and the ubiquitin–proteasome system [139]. More recently, with the advent of correlative super-resolution microscopy, dysmorphic organelles, vesicular structures, subtypes of lipid constituents, and membranous fragments surfaced as pivotal components of LBs [140, 141]. Furthermore, the authentication of neuropathological hallmarks and cytoskeletal proteins in LBs [142–145] essentially challenged the unresolved interactability and spatio-temporal dynamics of these heterogeneous inclusions. Tau protein, a microtubule-associated scaffolding element of the AD neurofibrillary tangle, was sporadically associated with a subset of LBs [146]. Likewise, the hyper phosphorylation of tau at Ser396 was discernible in synaptic-enriched fractions of DLB, AD, and PD brains [147], while α-synuclein genetic variability has been shown to modulate neurofibrillary tau pathology [148]. Overall, this posits that compensatory mechanisms may emerge in the face of concomitant α-synuclein and tau burden, which construes the frequent phenotypic overlap between DLB and AD.

## The α-synuclein architecture

An inherently disordered protein preponderantly disseminated in the brain [149, 150], the α-synuclein assimilated new-found relevance when its’ aberrant conformations nucleated the intracytoplasmic inclusion bodies of Lewy body diseases [25, 131]. Since a protein’s homology and configurational dynamics are coupled to its function, a concerted effort was subsequently initiated to designate the sequence and biophysical determinants that command α-synuclein’s proteome integrity and aberrant behavior. A highly divergent 14-kDa protein (140 amino acids) [151], the α-synuclein is defined by a lysine-rich membrane binding N-terminal amphipathic domain (residues 1–60), an aggregation indispensable central hydrophobic core (residues 61–95), and a disordered, acidic carboxy-terminal tail (residues 96–140) that is rich in proline, glutamate, and aspartate [152, 153]. The N terminus domain of α-synuclein comprises seven 11 residue imperfect tandem repeats, with a highly conserved central hexamer motif (KTKEGV) that wields an intrinsic tendency to approximately form two amphipathic alpha-helices (amino acids 3–37 and 45–92) in an antiparallel manner, flanking a truncated bridging region [152, 154–156]. By virtue of numerous repeats, the architectural flexibility of this domain enables the polypeptide to precisely potentiate three turns of the helix and establish contact preferentially with high curvature membranes [156–161]. Remarkably, the α-helical conformational conversion adopted upon membrane surface binding is stabilized by acidic phospholipid headgroups, signifying a synergistic interplay between the membrane and lysines positioned at opposing ends of the helix [156]. It is also riveting to note that all known genetic mutations affiliated with synucleinopathies—A53T, A53E, A30P, E46K, G51D, and H50Q are clustered within this domain [64, 162–168]. While these missense mutations, with the exemption of A30P, A53E, and G51D, have shown to escalate the tendency of α-synuclein to concoct morphologically insoluble discrete aggregates [164, 169–176], the bona fide mechanism through which these mutations prompt aggregation has not been convincingly presented. Nonetheless, the nonconservative E46K mutant is perhaps the only hereditary mutation that transpires in a clinical picture reminiscent of DLB since the clinical phenotype of E46K patients centered around severe and rapid disease evolvement alongside early-onset parkinsonism [64]. The crystalized hydrophobic central core of α-synuclein (residues 61–95), otherwise called the non-amyloid-β component of Alzheimer’s disease amyloid (NAC) [131, 151], encompasses two supplementary KTKEGV sequence motifs and a highly amyloidogenic segment that is accountable for the conformational plasticity of the protein [177]. Intriguingly, the deletion or disruption of sizable subdivisions within this domain immensely abrogated the oligomerization and fibrillogenesis of α-synuclein [178], accentuating the salience of NAC domains in the pathogenic cascade of α-synuclein aggregation [179–181] and its’ self-assembly into amyloid fibrils [182–185]. Originally projected to be a requisite for perpetuating the solubility of the protein, the C terminus of human α-synuclein (residues 96–140) contains 15 acidic amino acids (10 Glutamate and 5 Aspartate residues) and 5 Proline residues (P108, P117, P120, P128, and P138) that help to circumvent the spontaneous conglomeration of aggregation-prone conformations by masking the hydrophobic NAC domain [152, 154, 186, 187]. Owing to its higher proportion of charged residues and low hydrophobicity, it is conceivable that this domain undergoes specific phosphorylation at discrete sites [188, 189] and is responsible for the intrinsically unstructured topology of α-synuclein. As radical as it may seem, there is now compelling evidence that the obliteration of C terminus, in tandem with alterations to the domain hydrophobicity, may possibly compromise the membrane-binding machinery and enhance the aggregation propensity of α-synuclein [190–195].

## Understanding the biology of α-synuclein: membrane remodeling and synaptic behavior

**Fig. 1:**
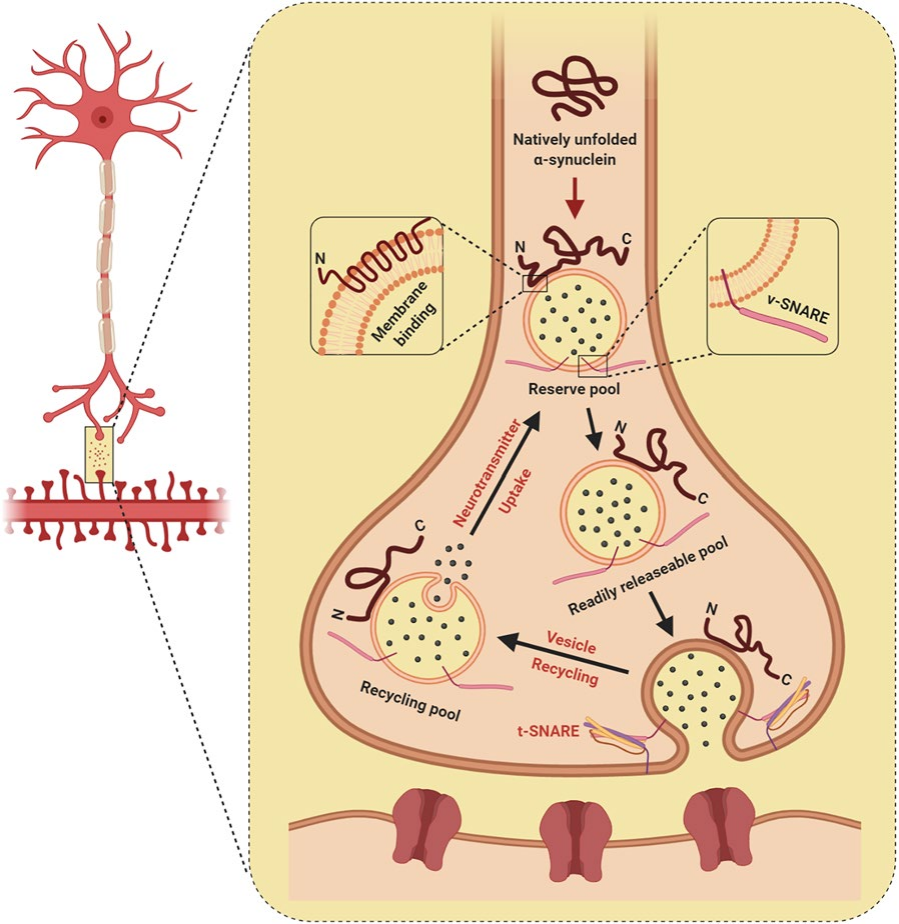
This schematic highlights the regulatory roles of α-synuclein in harmonizing synaptic homeostasis at the presynaptic terminal by coordinating vesicle trafficking, vesicle refilling, and the interactions between vesicle-associated SNARE (v-SNARE), membrane-associated SNARE (t-SNARE), and neurotransmitter release. Figures were created with Biorender.com

The presynaptic confinement of α-synuclein has become well entrenched ever since its discovery as a phosphoprotein that is vastly enhanced in synaptic boutons, which sprout from axons of diverse neurochemical phenotypes [154, 196, 197]. Yet, in spite of the initial affiliation with synaptic vesicles, α-synuclein is not consistently existent in all presynaptic terminals and curiously appears to be among the last proteins to approach the evolving synapse during synaptogenesis [198]. The discrepancies of these explications in neurodegenerative disorders have led to the fundamental question of how α-synuclein localizes to the biochemically distinct synaptic termini. Unswerving with its portrayal as a weakly associated peripheral membrane protein, it has been presumed that α-synuclein depends on the N-terminus domain to mediate membrane binding in cells [160, 174, 199, 200]. However, subcellular biochemical fractionation of brain tissue stipulates that an immense majority of synucleins behave almost entirely as soluble monomeric proteins, with an extremely weak affinity towards native synaptic vesicles [201–203]. In an endeavor to visually trace the dynamics and intrinsic mobility of α-synuclein in live intact cultured cells, hippocampal neurons derived from transgenic mice were transfected with GFP-labelled synuclein while individually isolated presynaptic boutons were exposed to photobleaching. Interestingly, the fluorescence recovery following photobleaching was persistently rapid, implying that the protein is exceedingly mobile [201]. On the contrary, when this experimental paradigm was extended to fluorescently-tagged human α-synuclein in individual cortical neurons of transgenic mice, signal recovery was prominently delayed and less exhaustive in synapses with increased synuclein expression, reputedly due to substantial in vivo aggregation [204]. Despite its greatly diminished tethering with cellular membranes, α-synuclein conversely recuperates more sluggishly than GFP after photobleaching [201], raising the plausibility that the N-terminal membrane-binding domain of α-synuclein may be spearheading the interplay. Supporting this notion, the A30P point mutation notably eliminates the synaptic enrichment of α-synuclein, obliterates the interaction of α-synuclein with artificial [205] and native biological membranes, and expedites fluorescence recovery to that of GFP following photobleaching [201, 206]. Amid these studies, counter-intuitive chemistry has alternatively described α-synuclein’s N-terminal region as a membrane curvature sensor, whose apparent “design defect” translates into an extraordinary ability to unambiguously adsorb to the surface of highly curved biological membranes (Fig. 1) [200, 207–209]. Accounting for this marked predilection is the hydrophobic fragment of the N-terminal amphipathic region, which harbors a sequence of threonines within the repeat that compromises the synergism of α-synuclein with membranes scrupulously as a means to attain specificity for high curvatures [210]. In conjunction with the aforementioned repertoire of membrane curvature dynamics, α-synuclein notoriously appears to possess a precise inclination towards the assemblies of lipid rafts, a membrane micro-domain that is negligible in fluidity but fortified with cholesterol and saturated acyl chains [201, 211–214]. Indeed, the A30P mutational derangement of lipid rafts prevented the agglomeration of α-synuclein in presynaptic boutons [201], complementing the relevance of this interaction for neurons. Recapitulating the specificity observed in vitro, biochemical methods have analogously detailed that α-synuclein necessitates a “mish-mash” of phospholipids with oleoyl and polyunsaturated acyl chains, signifying that it may irrevocably identify the phase boundary that ensues between membranes that vary in fluidity [215]. Remarkably, there was an irrefutable prerequisite for the acidic headgroup appertaining to polyunsaturated acyl in preference over the oleoyl chain, albeit the curvature-sensitive membrane tethering [215]. In accordance with this consideration, α-synuclein has been found to influence lipid packing within cholesterol-enriched raft-like domains [216], suggesting that α-synuclein is not only congregated by these structures but also effectuates their structural remodeling. Furthermore, α-synuclein has a notoriety for being implicated as a fatty acid-binding protein [217] that modulates the uptake of polyunsaturated fatty acids to catalyze the formation of highly soluble lipid-dependent oligomers [218–221]. Affirming a role for this phenomenon in α-synuclein deficient mice, astonishing changes were observed in acyl chain composition [222], while fatty acid uptake and metabolism turned out to be exaggerated [223–225]. Nonetheless, subtle alterations in acyl chain orientational order may modulate membrane fluidity and trafficking, and variations in α-synuclein expression level appear to augment clathrin-mediated endocytosis [226].

Notwithstanding the tremendous diversity in original publications, the dauntingly complex presynaptic enrichment of α-synuclein [197, 198] and its contiguous interaction with synaptic membranes have emphatically implied a regulatory role in the molecular machinery that mediates synaptic neurotransmission (Fig. 1) [193, 227–231]. Electrophysiological analysis of α-synuclein deficient mice has previously demonstrated a strikingly selective deterioration in hippocampal synaptic responses, following sustained high-frequency tetanic stimulations that exhaust the docked and reserve pool of synaptic vesicles [227, 232]. Concurrent with the altered responses, electron microscopic examination of mice lacking α-synuclein further displayed profound deficiencies in the rejuvenation of docked pools from the reserve pool. In a similar fashion, the repression of α-synuclein expression through antisense oligonucleotide pharmacokinetics prompts a significant diminishment in the attainability of reserve synaptic-vesicle pool in cultured hippocampal neurons [228]. On the basis of these observations, α-synuclein unexpectedly appears to orchestra the refilling and mobilization of synaptic vesicles from the reserve pool to the site of synaptic vesicular release. Perhaps not surprising, compelling support for this hypothesis came from transgenic model organisms and viral gene-delivery systems that were employed to over-express human α-synuclein, which elegantly demonstrated considerable deficits in synaptic vesicle exocytosis with diminutions in neurotransmitter release [233–238]. Contemporary ultrastructural paradigms subsequently publicized that the overabundance of α-synuclein precipitates a depletion in “readily releasable” vesicles [236] and physiologically disrupts the reclustering of synaptic vesicles following endocytosis, instigating a decrement in the synaptic vesicle recycling pool density [235]. Under similar circumstances, α-synuclein in overexpressing neurons unequivocally diminished dopamine reuptake in dopaminergic terminals [237] and impeded the intersynaptic vesicular dynamics between presynaptic boutons, resulting in a smaller reserve pool of vesicles [234]. The plausible portrayal of α-synuclein in harmonizing synaptic homeostasis, however, is not assiduously affiliated to its precise connectivity with synaptic vesicles. The multifarious α-synuclein has been shown to interact with synaptic proteins [239] that facilitate exocytic and synaptic vesicle motility, such as the phospholipase D2 and Rab GTPase-activating proteins [240, 241]. In this context, α-synuclein engaged as a tightly coordinated chaperone for the trimeric supramolecular SNARE complex by dramatically orchestrating the dissipation and coordinating the recruitment, safeguarding, and rigorous configuration of this multifaceted machinery [242]. These elucidations raise the possibility that α-synuclein dictates the trafficking of synaptic vesicles, regulation of vesicle exocytosis, and may theoretically mediate a more refined reciprocal phenomenon by domineering synaptic homeostasis-affiliated proteins (Fig. 1). The fact that synuclein deficient synapses are physiologically relevant connotes that synucleins are not integral denominators of the neurotransmitter release machinery but are redundantly essential for the long-term synchronization and maintenance of presynaptic efficacy [243]. Akin to the expunction of synucleins, the transgenic expression of α-synuclein in cysteine-string protein-α (CSPα)-ablated mice was able to abrogate progressive neurodegeneration [244], indicative of a neuroprotective role for α-synuclein in protecting nerve terminals against lethality. These observations appear to be mediated by a downstream mechanism that relies upon phospholipid binding, as the A30P α-synuclein mutant, which is greatly impaired in membrane binding, failed to rescue CSPα knockout mice from the deleterious consequences of CSPα deficiency.

## The α-synuclein alchemy: conformational heterogeneity and biological consequences

**Fig. 2:**
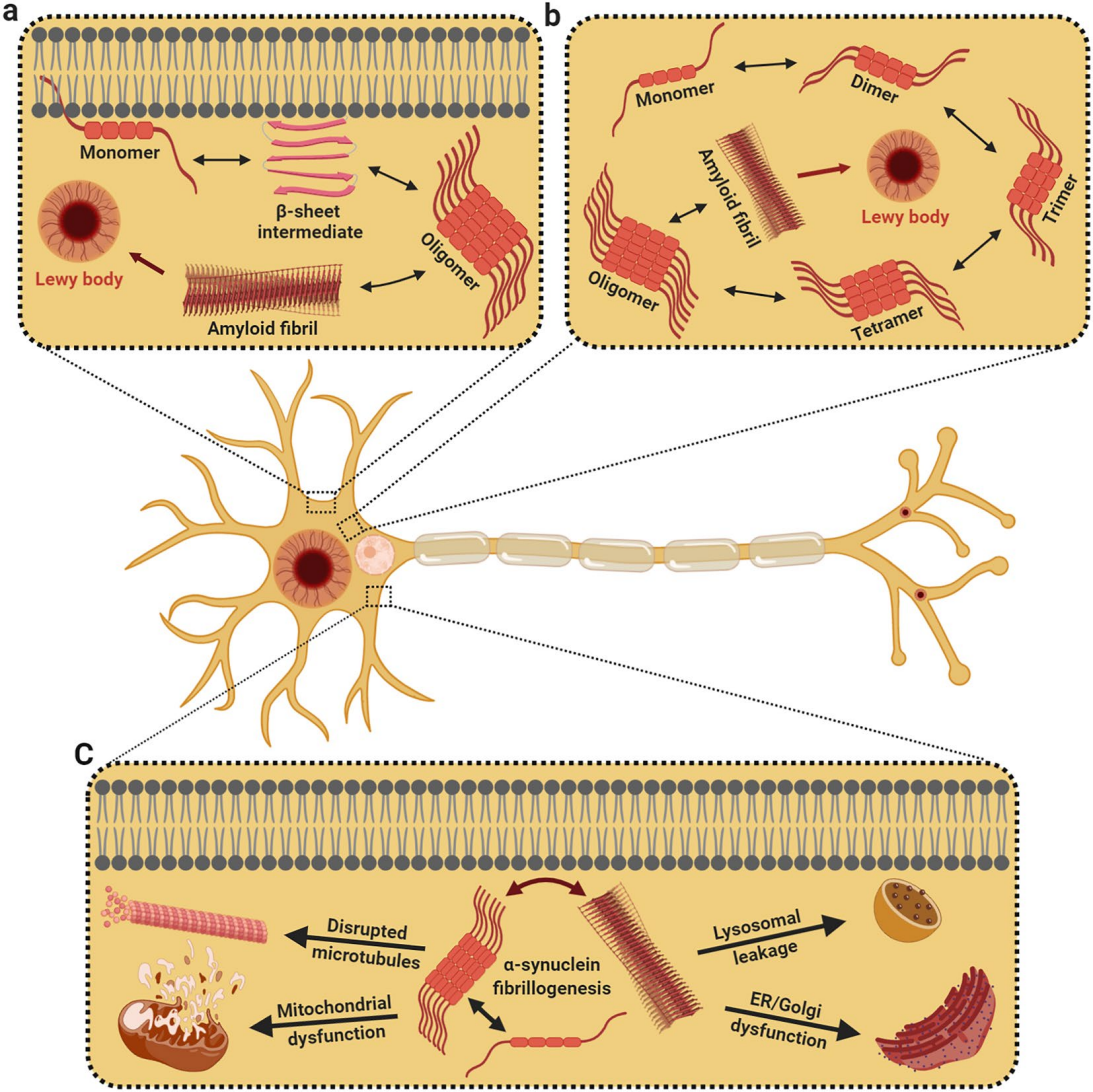
Under pathological circumstances, α-synuclein aggregation could potentially take place in affiliation with the cellular membrane or in the cytosol. **a** Membrane-bound monomeric α-synuclein assumes an α-helical structure, but at elevated levels, the monomer endures conformational change to generate membrane-bound β-sheet structures that self-associate to form oligomers and fibrils. **b** In the cytoplasm, unfolded monomers fluctuate through conformational space to form unstable dimers, which undergo reorganization to generate oligomers of varying morphologies that eventually transform into fibrils. The erratic accretion of these fibrils leads to the amassing of intracytoplasmic Lewy bodies. **c** During α-synuclein fibrillogenesis, oligomers and amyloid fibrils are immensely noxious, compromising microtubule dynamics, endoplasmic reticulum–Golgi trafficking, and mitochondrial function. Figures were created with Biorender.com

Over the last decennary, tangible evidence suggests that α-synuclein is able to stochastically fluctuate through conformational space and undergo dramatic internal reorganization from their primitive states to form intermolecular β-sheet-rich entities; ranging from monomers, tetramers, and higher-order soluble oligomers to large insoluble fibrillar polymorphs (Fig. 2a, b) [245–250]. The abstruse heterogeneity and rapid interconversion tendency to assume higher-order aggregates, however, have made it exceedingly laborious to reconcile high-resolution structural details for the native assembly and naturally-occurring multimeric conformations. Years of elegant work have previously demonstrated that recombinant α-synuclein isolated from bacterial expression systems, under native or denaturing environments, subsists arbitrarily as stable unfolded monomers with insubstantial secondary structural features [153, 245, 251–253]. This ostensibly anomalous behavior was further ratified by the analysis of purified α-synuclein, which revealed a compact monomeric state for native α-synuclein that counteracts spontaneous assemblage, arrogates α-helical conformation upon phospholipid tethering, and withstands conformational metamorphoses prior to oligomerization and fibrillogenesis [254, 255]. While these annotations stipulated that native α-synuclein is rudimentarily an ill-defined monomer that adopts multiple forms of compact conformers depending on its cellular localization and membrane interactions, a lingering ambiguity endured concerning the apparent native size of the protein. Consequently, further investigations on the α-synuclein native state were reignited several years later, and this time, suggesting that α-synuclein behaves intrinsically as dynamic α-helical tetramers that are impervious to aggregation and fibril assembly [256–258]. In parallel, complementary biophysical efforts by several groups insinuated that only a small proportion of α-synuclein assembles into α-helical trimers and tetramers while the majority of species perseverated as deranged monomers [257, 259, 260]. Concertedly, these illustrations sparked off an alleged controversy and effectuated a reappraisal of the native state of α-synuclein. Even though synonymous inquisitions in the mouse brain reaffirmed that the predominant native form of α-synuclein is an inherently disordered monomer with minimal secondary structure [246, 261], conformationally diverse α-synuclein multimers were also present in post-mortem brain tissue [251]. This continuum of conformational states, embraced by native α-synuclein, evolved through a series of equilibria that adopted distinct assemblies under precise stress-induced constraints or upon interaction with specific lipids, ligands, proteins, or biological membranes [161, 168, 251, 262–265]. Considering the heterogeneity and diversity of oligomeric variants that were of disease relevance [69, 266–271], numerous propositions have accordingly explored the molecular and structural determinants that influence α-synuclein’s initial facile oligomerization. Compellingly, mildly acidic milieus [272], low fractions of negatively charged lipids [273], and specific post-translational modifications [189, 274–277] regulate α-synuclein oligomerization. Besides, α-synuclein interacts with polyunsaturated fatty acids [220], lipid vesicles [278], transition metals (Fe^2+^, Cu^2+^, and Zn^2+^) [254, 276, 279], and drug-like molecules such as dopamine [280] to promote its’ self-assembly and formation into β-sheet rich soluble aggregates. Upon assembly, α-synuclein oligomers sequentially undergo conformational switching to become proteinase-K-resistant oligomeric precursors that generate significant levels of oxidative stress before metamorphosing into downstream fibrils [281]. These oligomeric states of α-synuclein may chemically permutate in equilibrium, remodeling their mechanical behavior, either engaging as on-pathway intermediates or kinetically trapped assemblies from which fibrillation is no longer conducive [282].

In spite of the wealth of empirical data, there is no unanimity on whether oligomers or downstream fibrils are the more noxious entity. Fastidious observations of human α-synuclein-expressing mesencephalic neurons suggested that oligomers are more noxious since α-synuclein purified from DLB brain extracts displayed escalated levels of soluble, lipid-dependent α-synuclein oligomers [220]. In a similar vein, the pioneering characterization of oligomeric landscape in post-mortem DLB brain homogenates validated the existence of soluble α-synuclein species that exacerbated phospholipid membrane integrity [69, 271]. The diagnostic utility of cerebrospinal fluid (CSF) α-synuclein species to distinguish DLB patients from AD individuals further reported a unique trend of high-oligomeric-α-synuclein CSF levels, albeit a lack of correlation with cognitive performance [270]. Conversely, while exosomal α-synuclein levels correlated with the severity of cognitive impairment in DLB patients, isolated DLB-patient CSF-derived exosomes governed the oligomerization of soluble α-synuclein in recipient cells [283]. Intriguingly, the inoculation of DLB brain-derived exosomes into healthy rodent brain tissue was sufficient to propagate α-synuclein aggregation, bolstering the hypothesis that pathogenic α-synuclein oligomers may be preferentially sorted into exosomes to facilitate α-synuclein fibril growth [284]. The adaptation of an in vitro amplification technique, designated “real-time quaking-induced conversion (RT-QUIC),” jointly implied that the oligomeric forms of α-synuclein are the seeding species that cause prion-like propagation [285]. Coherent with these findings, α-synuclein-associated mutations (A53T and A30P) accelerated oligomerization, not fibrillization [286], while stereotaxic inoculation of oligomer-forming rather than fibril-promoting α-synuclein variants induced severe in vivo deterioration [287]. Additionally, much evidence has been adduced to suggest that α-synuclein oligomers may dysregulate cell function and culminate in synaptotoxicity [252, 286, 288] by eliciting lysosomal leakage [289], compromising mitochondrial activity [290, 291], dishevelling microtubule dynamics [292], and physically altering the axonal transport of presynaptic proteins [234] (Fig. 2c). While it is conceivable that α-synuclein toxicity involves distinct intermediates on the pathway en route to fibril formation, a series of kinetic signatures insinuate that the process involving the conversion of oligomers to fibrils itself contributes to α-synuclein-mediated neurodegeneration. Expression of α-synuclein mutants designed to promote fibril formation propensity (S129A) was toxic to rat models [293, 294], whereas mutations that impede α-synuclein oligomerization and fibrillogenesis (S87E) catalyze considerably less α-synuclein aggregation [274]. Likewise, complementary expression of artificial mutant variants of α-synuclein (E57K and E35K) resulted in conformationally snared oligomers that were profoundly noxious [288] to dopaminergic neurons in animal models of synucleinopathies [287]. Conjointly, mutations that augment α-synuclein oligomerization unveiled high neurotoxicity, but a sustained progressive loss of dopaminergic neurons was undisputedly reliant on the potential of α-synuclein variants to form fibrils [295]. As such, one may presume that the fibrillary α-synuclein assemblies might be significantly more pathogenic than their precursor on-assembly pathway oligomers [176, 296] since the nigral inoculation of α-synuclein fibrils aggravated motor deficits and induced more pronounced Lewy body/Lewy neurite-like inclusions [297]. In support of this proposition, the Lewy-associated filamentous α-synuclein enriched in detergent-insoluble fractions of DLB patients projected the ability to alter neuritic outgrowth in human iPSC-derived neurons [69]. The revelation of α-synuclein assemblies with distinct strains of pathological fibrils further led to the premise that the fibrillar polymorphs display differential seeding and fibril-templating efficiencies that may account for the heterogeneous neurotoxic phenotypes in DLB [176, 298, 299]. As innocuous as it seems, the structural rationale underlying fibril polymorphism is that the α-synuclein fibrils are made up of two intertwining protofilaments, and the discrepancies in the assembly of these protofilaments may give rise to discrete conformational polymorphs [300, 301]. Nevertheless, it remains obscure if preformed fibrils which maturate in LBs may instigate disease-specific cytopathology. Traditionally, whilst the omnipresence of cortical LB densities are pathognomonic for cognitive dysfunctions [24], there is limited evidence to reinforce a correlative relationship between LB burden and the severity of dementing phenotypes [27, 88, 108, 302]. In the course of addressing the malleability and dynamism of LB composition with respect to the lack of overt phenotype, key pathological events were often reported to be imperiled and overwhelmed prior to LB biogenesis. These cellular insults include synaptic collapse [40], reduced neurofilament mRNA levels [303], the amassing of specialized axonal proteins [304], and the implementation of apoptotic signaling cascades [305]. With these caveats in mind, it is tempting to speculate that the acquisition of deleterious hallmarks is instead galvanized by ill-defined heterogeneous oligomers. Taken together, the mechanistic relationship between oligomers and fibrils remains to be clarified since a well-founded conjecture is complicated by the heterogeneity of diverse experimentally observed states. Despite the fact that oligomers are conceivably implicated in the breakdown of neuronal homeostasis, the stable protuberant nature of fibrillar α-synuclein assemblies appears to be the most competent at propagating itself both in vitro and in vivo. The inconvenient truth is that the “oligomer” and “fibril” nomenclatures lacked the fidelity and mechanistic precision required for the critical appraisal of physiological entities. Rather, numerous conformations of these assemblies exist, which commands their biophysical profile, and may account for unique strains of polymorphic aggregates resulting in distinct pathobiological traits [298, 306].

## Beyond α-synuclein propagation: from transmission to DLB pathogenesis

**Fig. 3:**
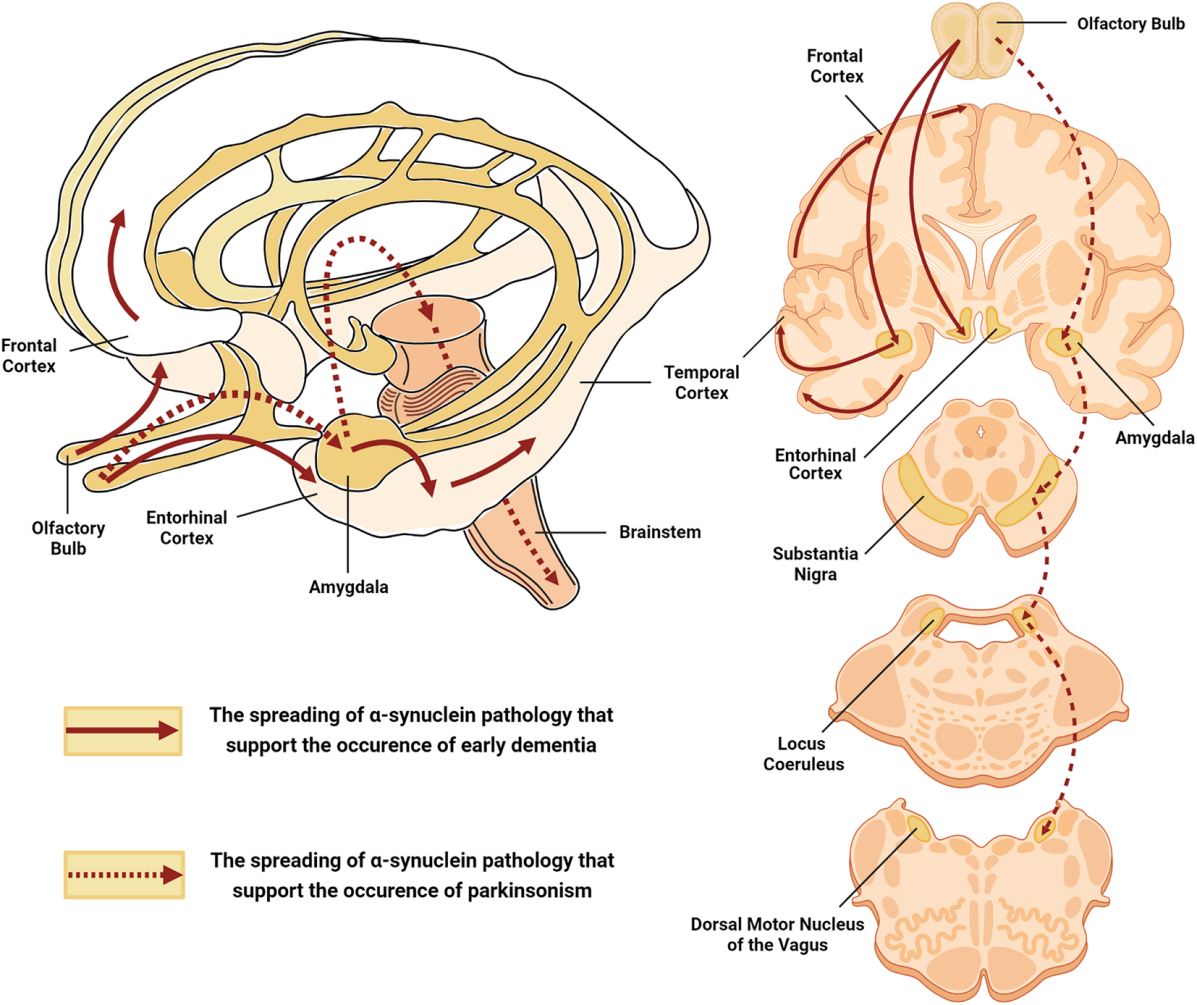
Schematic representation of the hypothetical DLB staging construct with stereotyped incursions of α-synuclein pathology across limbic and neocortical networks that might support the occurrence of early dementia with parkinsonism. Figures were created with Biorender.com

The central tenet of presynaptic α-synuclein fidelity, under physiological circumstances, is that the α-synuclein is an intracellular synaptic protein that coalesces with vesicles [197, 231]. Yet, under distinct pathological milieus, parsimonious explanations theorized that the toxic oligomeric species of α-synuclein could be eliminated from neuronal cells through unorthodox secretory pathways [307–310]. As such, perturbation of the intracytoplasmic degradation pathways, for instance, the autophagic signaling network [311, 312], might jeopardize and catapult the pathological release of α-synuclein in degenerating neurons [313, 314]. In principle, the sequestration of extracellular α-synuclein oligomers entails unconventional exocytosis in clear-core synaptic vesicles [313, 315], exosome-mediated discharge [283, 308, 316–318], and infiltration from the donor membrane [319, 320]. Circumstantially, these pathogenic α-synuclein aggregates are unequivocal in substantiating trans-synaptic and intracellular transmissibility through the neuraxis [308, 313, 321], where they govern intraneuronal aggregation [322] and are ‘primed’ to exacerbate neuroinflammation [323–326]. Exemplifying the molecular basis of this stereotyped spread, various models of Lewy-prone systems have displayed robust induction of α-synuclein inclusion pathology [327] in human neuronal precursor grafts and human fetal grafted neurons [323, 328–332]. Currently, there are several theoretically acclaimed neuropathological grading paradigms to evaluate the topographic trajectory of α-synuclein, entailing a semiquantitative staging of α-synuclein to address the chronological severity in discrete brain regions. The hierarchical caudo-rostral dissemination of Lewy body-related pathology in LBD, systematically proposed by Heiko Braak and Kelly Del Tredici, has been spatiotemporally interpreted to corroborate a cohesive hypothesis of unidirectional α-synuclein distribution through specific pre-established neuroanatomical circuits [120, 333–335]. In an influential series of histopathological evaluations, the provocative “Braak hypothesis” first outlined that the dorsal motor nucleus of the vagus nerve, and to a lesser extent, the olfactory system, serve as peripheral entry points for the α-synucleinopathy (Stage 1). Essentially, these misfolded versions of α-synuclein travel transneuronally in a stereotypic fashion across the medulla and pontine tegmentum (Stage 2), imprinting the retention of α-synuclein in somal Lewy inclusions and provoking the polymerization of neighboring synuclein deposits in the midbrain and amygdala (Stage 3). It then follows that the α-synuclein transcellularly deposits deeper into the temporal cortex (Stage 4) and neocortex (Stages 5 and 6), exposing the cardinal cognitive deficits observed in LBD [106, 120, 333–335]. Suffice it to say here, that in DLB, which at first manifests with clinical dementia and only sporadically with extrapyramidal signs, this topographical α-synuclein-centric expansion map is not favorable. Overall, though, it was consequently reasoned that α-synuclein pathology might initially transpire in neocortical and limbic areas. Marui et al. [41], in their seminal investigation of the neuroanatomic contiguity of DLB pathology, explored the regional focality of Lewy-related α-synuclein dissemination in the brain of cases that fulfilled the clinicopathological diagnostic criteria for DLB. Based essentially on topographic proximity, the brain regions reviewed encompassed the hippocampus (CA2/CA3), the amygdala (cortical and accessory basal nuclei), and the superior frontal, middle temporal, insular, transentorhinal, and entorhinal cortices. Compellingly, the widespread evolution of Lewy pathology defined exclusively by a morphological staging scheme engaged the amygdala first, followed by various limbic regions, most notably the transentorhinal, insular, entorhinal, and CA2/3 cortices, and, ultimately, the neocortex [41]. But even so, one fundamental drawback of this framework was that the olfactory bulb, which is implicated in virtually all of the DLB cases, was not cross-examined [336]. This forestalled the feasibility of drawing firm inferences about how the olfactory bulb proteotype might hypothetically tailor into such a staging template. Nevertheless, these findings are particularly consequential since they disclosed that olfactory and limbic structures are most susceptible and succumb early in the disease process. Among the many amygdaloid subnuclei, the cortical areas are the ones that receive projections directly from the olfactory bulb [337, 338]. These evaluations have led to the assumption that the distribution of Lewy pathology in the amygdala of DLB cases mirrors the input from the olfactory bulb. Yet, the predictive validity of this staging scheme is ambiguous, considering there was a lack of correlation between the α-synuclein stages and the clinical severity of dementia and psychiatric comorbidities. According to these considerations, it is inferable that the pathoarchitectonic pattern of DLB is not just confined to the systematic progression of α-synuclein pathology but also to the subsequent influence of more-deterministic AD-relevant pathologies in anatomically related foci [339]. Consistent with this supposition, Rey et al. [340] unveiled unprecedented insights into the spatiotemporal pattern and transneuronal propagation of pathologic α-synuclein from the olfactory bulb. In a notable series of experiments, the authors inoculated recombinant α-synuclein fibrils into the olfactory bulb of wild-type mice and probed their transmissibility through the neural circuitry. Within 1-month post-inoculation, α-synuclein pathology was preliminarily disseminated in the ipsilateral entorhinal and piriform cortices, the cortical amygdaloid nuclei, and the contralateral and ipsilateral anterior olfactory nuclei. By 3 months, α-synuclein-positive inclusions have progressively spread sequentially to the hippocampus, the basal amygdaloid nuclei, and the insular, ectorhinal, and orbitofrontal cortices. At 6 months, the spatial spreading of α-synuclein aggregates evolved considerably to the nucleus of the lateral olfactory tract, the olfactory tubercle, the central amygdala, the dentate gyrus, the CA2/CA3 fields of the hippocampus, the thalamus, and the hypothalamus. Eventually, by 12 months post-inoculation, the seeded α-synuclein inclusions were able to serially propagate to the associative neocortex, the locus coeruleus, the substantia nigra, and the raphe nuclei [340]. Despite assertions that the pathogenic templating of endogenous α-synuclein amass in a connectome-dependent manner, there remains an unsatisfying disconnect between α-synuclein deposits and its subsequent engagement with additional brain regions 2 years post-inoculation [341]. The shortcoming fueled speculation that transmission of α-synuclein pathology is likely to be perturbed by premature α-synuclein-induced neuronal loss, which compromises the neural circuit integrity, or by proteolytic mechanisms that facilitate α-synuclein degradation. This notion has gained traction since no α-synuclein-positive inclusions were disseminated in mouse brains 21 months post-nasal inoculation of DLB-associated α-synuclein fibrils, further insinuating that α-synuclein aggregates cannot possibly navigate through the nasal mucosa [342]. Tantalizing as it may be, although dense α-synucleinopathy expanded beyond the limbic and associative neocortical connectomes, distinctive inclusion bodies were only ‘occasionally’ concentrated in the substantia nigra [340]. One might connote that in terms of clinical correlates, such template-directed sequential dispersal of Lewy pathology would likely be associated with early-onset dementia and subsequent parkinsonism, mimicking the clinical phenotype of DLB (Fig. 3). In this structure-oriented view, the iatrogenic transmission of α-synuclein pathology from the olfactory bulb is able to extend beyond the olfactory limbic axis and substantially infiltrate the associative neocortex [340]. Of paramount importance, the preponderance of cortical amygdaloid-predominant α-synuclein pathology originated from the olfactory bulb during the earliest stage of spatial dissemination. Similarly, the proposed stereotypical expansion of incidental LB pathology by Marui et al. [41] acknowledged that the cortical nuclei of the amygdala were indeed the primary site in which α-synuclein inclusions are first discerned. Together, these directional predilections buttress the conjecture that, in DLB, the cortical zone of the amygdala may represent a preferential locus for α-synuclein deposition wherein pathologic aggregates are reached via the olfactory bulb. If this corollary is valid, it raises the intriguing possibility that the translation of this discernible gradient in regional hierarchy into a unidirectional chronological sequence may provide the basis for a hypothetical DLB staging construct (Fig. 3). In the same way that a caudorostral trajectory subsists in PD, a predefined nasal route with stereotyped incursions of the α-synuclein pathology across limbic and neocortical networks could also be conceivable in DLB. This spatiotemporal axis, proposed by the aforementioned conceptualization, would presumably result in a clinical picture in which overt cognitive deficits appear early, thus bringing forth a phenotype resembling DLB. Although mechanistically distinguishable, auxiliary determinants such as concomitant AD-type pathology may, in part, exacerbate the corruptive templating and DLB-specific spatiotemporal progression of α-synuclein lesions [339]. These, in turn, may influence the complementarity of molecular surfaces that interact to instigate the seeding cascade of afflicted areas, rendering certain brain regions selectively susceptible to further abnormal protein deposition. Moreover, the revelation of α-synuclein-driven phenotypic diversity in pathology is contingent upon neurons that are already burdened with concomitant Aβ- and tau-mediated pathological insults. In a self-perpetuating process, these subsets of compromised neurons tend to engage as trigger sites that synergize with lesioned assemblies to precipitate synuclein-based deposit accumulation in the neocortex. The concurrent manifestation of hyperphosphorylated tau, Aβ, and α-synuclein inclusions in the broad sense thus revamps the topographical accrual of anomalous proteinaceous entities and thereby influences the nature of the ensuing disease [339].

To this end, the perpetual mechanisms ascribed to the imminent spreading of extracellular α-synuclein oligomers have been warranted by endocytosis-mediated access [313, 343, 344], transmembrane penetration [158, 319, 345], trans-synaptic transmission [346, 347], and membrane receptor internalization [313, 344, 348]. Once within the recipient cell, internalized α-synuclein oligomers incapacitate the recipient cell by engaging as a molecular blueprint that further impels the morphogenesis of intracellular Lewy body-like aggregates [179, 324]. In fact, the topology of these induced inclusions argues in favor of a rate-limiting nucleation mechanism that pre-eminently heightens the cross-linking of assembly-competent oligomers, which is redirected by cooperative oligomeric growth and exponential fibril elongation by monomeric addition [272, 278, 349]. At the right stoichiometry, the binary switch between nucleation and elongation necessitates disordered oligomeric intermediates to adopt dynamically ordered configurations that are impervious to devolution and capable of evoking further fibrillation [350]. Such fine-tuning of fibrillation, in turn, can be catalyzed by α-synuclein-harboring familial mutants [351, 352] and posttranslational modifications such as truncation [353], phosphorylation [55], oxidation [354], nitration [355], glycation [356], and acetylation [357]. On this basis, the presence of seeding-competent fibrils can override the initial lag period of the primary nucleation phase [272], resulting in de novo secondary nucleation, which rudimentarily facilitates the generation of new entities on the surface of existing fibrils [358]. In analogy to mammalian prions, the so-called “nucleation-dependent polymerization” is usually invoked to illustrate the perplexing sequelae of intracellular oligomer and fibril propagation; and is thought to encipher the evolving neuroanatomical spread of α-synuclein pathology. This catastrophic assembly has been observed in a cell-based construct in which the inoculation of recombinant α-synuclein fibrils channeled the engagement of endogenous α-synuclein and self-amplification of Lewy body-like inclusions [179, 324]. In a similar fashion, the introduction of brain homogenates comprising α-synuclein protofibrils and fibrils considerably enhanced α-synuclein pathology and propagation in genetically engineered α-synuclein mice [359, 360]. Indeed, the suggestion that α-synuclein may propagate like a prion is entering a whole new realm of clinical relevance, given that it could unravel the connectomic distribution of Lewy pathology and aetiological heterogeneity across DLB. Regardless, the template-mediated prion-like amplification of pathogenic α-synuclein doesn’t appear to be solely defined by the ‘nearest neighbor’ rule or the strength of neuroanatomic connectivity. The engagement of neuronally expressed lymphocyte-activation gene 3 (LAG-3) demonstrated a high binding affinity towards preformed α-synuclein fibrils that initiated endocytosis from the extracellular milieu. What is more, the knockdown of LAG-3 substantially attenuated the cellular uptake of α-synuclein fibrils, diminishing the pathology set in motion [361]. Building on this finding, extracellular α-synuclein oligomers preferentially interact with the prion protein, eliciting a wide repertoire of signaling cascades that culminates with neuronal dysfunction [362]. Hence, according to these interpretations, the network signatures corresponding to the robust development of Lewy-type pathology may be actuated by cell- or region-autonomous mechanisms. Along the same line of thought, a low regional expression of native α-synuclein has been attributed to sequentially vulnerable brain areas that do not develop Lewy body-like α-synuclein inclusions [363] while attenuated cellular expression was prohibitive to intracellular aggregation [364]. In this regard, lower expression levels of physiological α-synuclein within distinct neuronal subpopulations may hinder the consequent amassing of intracellular aggregates by constraining the initiation nucleation phase in pathology-laden brains. Conceptually, these observations may represent a far end of a continuum that begins with extracellular α-synuclein seeds that presumptively choreographs specific patterns of neuronal vulnerability in a prion-like fashion. While transcellular propagation in vivo remains conjecture, such experimental systems necessitate rigorous substantiation, preferably by the observation that α-synuclein spontaneously disseminate aggregation through pre-existing neural networks.

## Concluding remarks

A blurring of the traditional distinction between physiologically relevant α-synuclein and its robust cytopathological signature in the DLB brain has heightened unwarranted apprehension over the neuropathological transmissibility of α-synuclein. The unsalutary intersection of disease-linked determinants that accelerate α-synuclein oligomerization has obscured the precise role of α-synuclein oligomers, fibrils, and prion-like α-synuclein propagation in the etiopathogenesis of DLB. Expanding the experimental momentum from genetically engineered systems to the DLB brain's neural circuits continues to be a constraining facet and mandates the exploitation of in vivo culture models derived from α-synuclein-rich brain extracts. The mechanistic commonality geared towards abating α-synuclein synthesis, toxicity, and aggregation are currently being scrutinized in preclinical models and lend credence to the establishment of mechanism-based therapeutics in DLB. We believe that more emphasis on unraveling the molecular mechanisms that unify the self-templating capacity of α-synuclein will substantially broaden our understanding of the pathocascades leading to neurodegenerative dementias such as DLB.

## Data Availability

Not applicable.

## Abbreviations

DLB: Dementia with Lewy bodies
PD: Parkinson’s disease
SDLT: Senile dementia of Lewy body type
AD: Alzheimer’s disease
PDD: Parkinson’s disease dementia
LBs: Lewy bodies
LB: Lewy body
LNs: Lewy neurites
NAC: Non-amyloid-β component of Alzheimer’s disease amyloid
GFP: Green Fluorescent Protein
SNARE: Soluble NSF Attachment Protein Receptor
CSPα: Cysteine-string protein-α
LBD: Lewy body dementias
LAG-3: Lymphocyte-activation gene 3
